# Mesoporous silica based recyclable probe for colorimetric detection and separation of ppb level Hg^2+^ from aqueous medium

**DOI:** 10.1038/s41598-019-55910-4

**Published:** 2019-12-18

**Authors:** Trisha Das, Debdas Singha, Ananya Pal, Mahasweta Nandi

**Affiliations:** Integrated Science Education and Research Centre, Siksha Bhavana, Visva-Bharati, Santiniketan 731235 India

**Keywords:** Environmental social sciences, Materials science, Nanoscience and technology

## Abstract

Functional mesoporous silica probes, **MCM-TFM** and **SBA-TFM**, have been synthesized with varying pore sizes and having S-donor sites judiciously selected to bind soft metal centers. The soft S-donor centers are contributed by the thiol functional groups that are introduced into the silica matrices by functionalization with *tris*(4-formylphenyl)amine followed by 2-aminothiophenol. The materials rapidly and selectively detect Hg^2+^ colorimetrically and the change in color profile can be perceived through bare eyes. The probes can decontaminate the pollutant heavy metal from aqueous medium at ppb level and the materials are recyclable and reusable for several separation cycles.

## Introduction

Mercury is a bio-accumulative, prevalent pollutant heavy metal which is found in air, water and soil^1^. It is not at all essential to the biological system. Its consumption can lead to several developmental delays and health problems that can damage the brain, nervous system, kidneys and endocrine system^2^. Mercury is released in the environment through conventional sources including natural and *anthropogenic* activities; as industrial wastes of various processes such as chemicals, electronic materials, batteries and fossil fuel combustion^3^. Apart from the elemental form, the most stable and common form of inorganic mercury, Hg^2+^ ion, is available in surface water due to its high solubility. This water soluble divalent mercury ion shows pronounced toxicity due to its strong affinity towards thiol (-SH) groups found in proteins and enzymes. Again, Hg^2+^ ion can transform to organic methylmercury (CH_3_Hg^+^), which can easily enter into the food chain. All forms of mercury, directly or indirectly, are threatening to public health and environment even at very low concentration. Thus, there has been a global agreement spurring research which aims to reduce mercury burden by removal and recovery of mercury ions from industrial waste water^4^. To protect the ecosystem, the permissible limit for the discharge of the toxic metal in aquatic systems is less than 1 ppb as per WHO guidelines^5^.

Considerable efforts have been made to develop new technologies for effective detection and removal of mercury from aqueous system^6–8^. Different types of small molecules have been reported to act as fluorescent sensors for selective detection of the metal ion^9^. On the other hand, various adsorbents have shown great promise for removal of the toxic metal due to cost-effectiveness of adsorption technology and simplicity of adsorption process in water purification. Common adsorbents like activated carbons^10^, zeolites^11^ and clays^12^ generally have low capacity and bind weakly to the heavy metal ion and thus thio-functionalized materials are more effective for Hg^2+^ removal due to soft-soft interactions^13,14^. However, most of the methodologies developed can either perform the detection^15^ or the scavenging task separately^16^, severely limiting their practical application. Moreover, the existing adsorbents suffer from drawbacks like poor surface area and inhomogeneous distribution of binding sites which affect the efficiency of the removal process. In this respect, mesoporous silica based structures have drawn considerable interest as they can simultaneously detect as well as separate the metal ions^17–19^ by virtue of the organic functional groups homogeneously anchored on their surface and large surface area with uniform pore size distribution. Multifunctional recyclable magnetic mesoporous silica^20^ based on CdTe quantum dots and rhodamine-6G has been reported for ratiometric sensing, adsorption and removal of Hg^2+^ ions. Thiol functionalized short channel^21^ SBA-15 and hollow mesoporous silica microspheres with magnetic cores^22^ have been used for the adsorption of Hg^2+^ ions. Di-thio grafted magnetic mesoporous silica nanoparticles^23^ proved to be highly efficient for removal of Hg^2+^ ions from environmental water samples.

**Figure 1:**
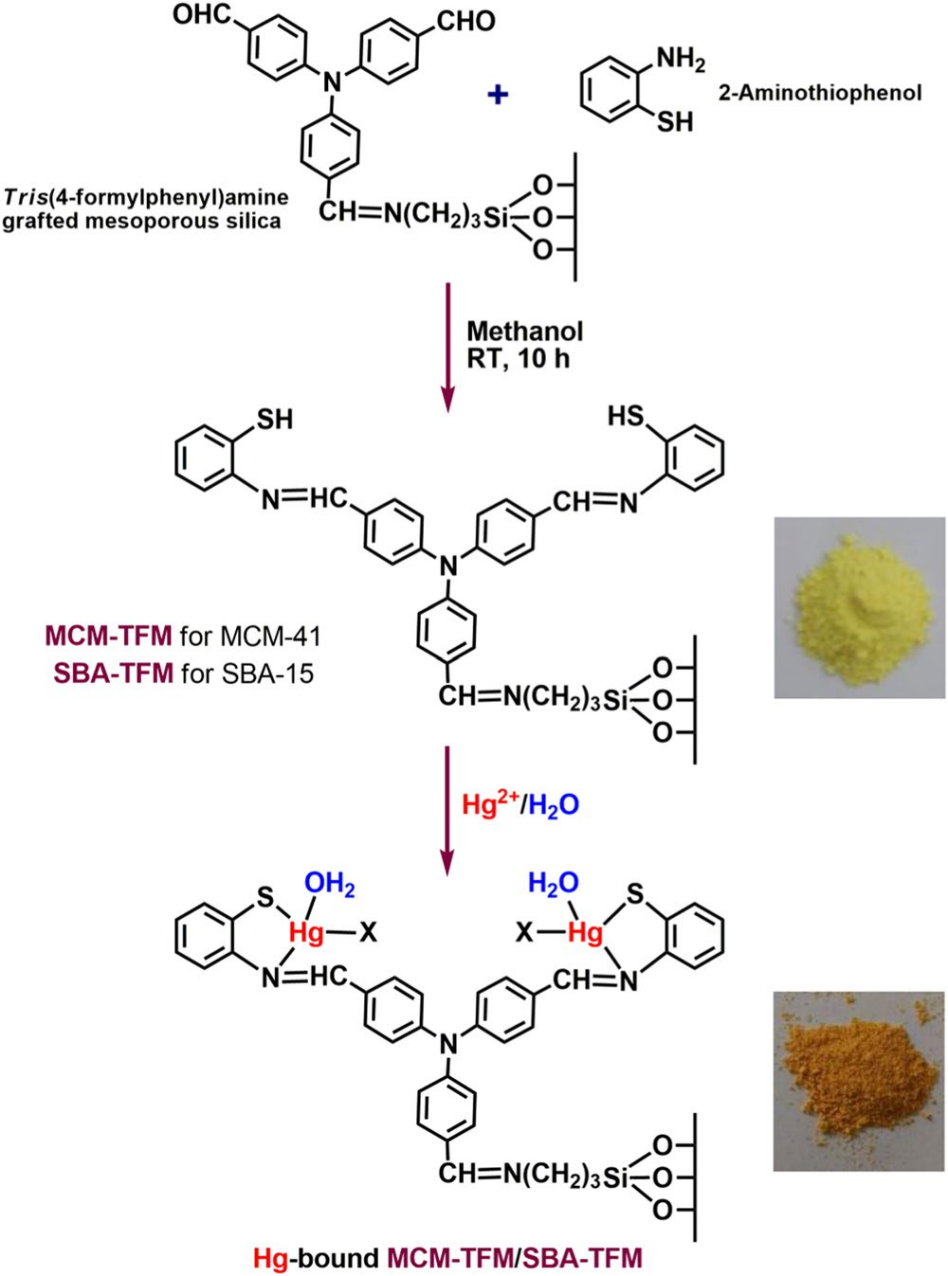
Syntheses of **MCM-TFM** and **SBA-TFM**.

In order to address the aforementioned challenges, in this contribution we report mesoporous silica, MCM-41 and SBA-15, based functionalized probes with S and N donor sites, **MCM-TFM** and **SBA-TFM** (Fig. 1), which can selectively sense (visual and spectrophotometric) as well as scavenge Hg^2+^ ions from aqueous medium through covalent interaction with very high efficiency. MCM-41 and SBA-15 have been chosen here as silica supports as they represent two distinctly different set of mesoporous structures, in terms of pore size and surface area and have been widely studied for various applications. Other silica supports have similar structures; for example, MSU-H is analogous to SBA-15^24^, and are expected to behave similarly. Characteristics such as high surface area, uniform pore structure and tuneable functionality are the advantages of such mesoporous silica based solid supports^25^. In addition to this, post-synthetic grafting of the matrices under homogeneous condition affords mesoporous silica nanocomposites with even distribution of the active functional groups. The thiol functionalized probes that have been prepared here show enhanced selective chromogenic behaviour toward Hg^2+^ ions and the colour change from pale yellow to dark orange is easily visible to the naked eyes.

## Results and Discussion

Two different silica supports, MCM-41 and SBA-15, which differ in their pore sizes, have been used here for the syntheses of S and N functionalized chromogenic detectors and scavengers of Hg^2+^ ion, **MCM-TFM** and **SBA-TFM** (Fig. 1), respectively. The trialdehyde unit present in the probes act as the chromophoric group^26^ that brings about the visual colour change whereas the S and N donor pockets trap the heavy metal. The aim here is to enhance metal removal efficiency which can be achieved by increasing the number of binding pockets. In this respect, the trialdehyde plays an important role by engaging one of its -CHO groups to bind to the silica support leaving the other two available to react with 2-aminothiophenol thus creating two donor pockets for binding Hg^2+^ ions per molecule of the trialdehyde.

### Powder X-ray diffraction studies

**Figure 2:**
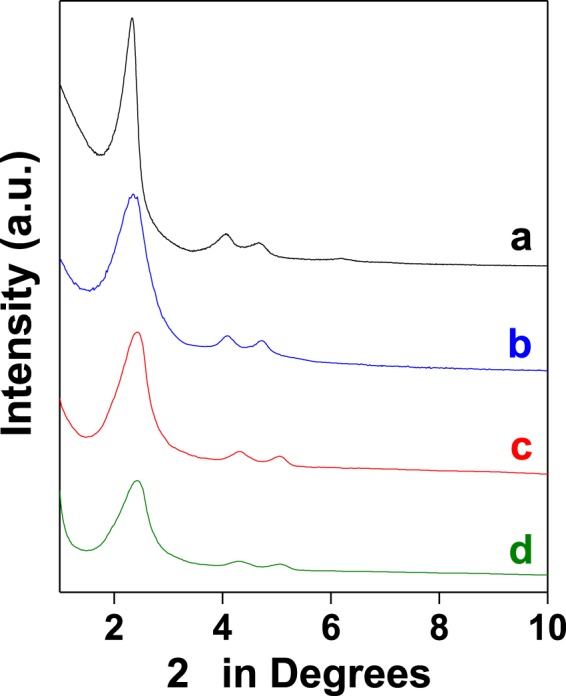
Powder X-ray diffraction patterns of (**a**) calcined MCM-41, (**b**) 3-APTES functionalized MCM-41, (**c**) *tris*(4-formylphenyl)amine loaded MCM-41 and (**d**) **MCM-TFM**.

The microstructure of the set of samples prepared over two different types of silica supports, MCM-41 and SBA-15, has been studied using powder X-ray diffraction. Two set of data has been obtained corresponding to MCM-41 and SBA-15 series of samples. The results for calcined MCM-41, MCM-41 functionalized with 3-APTES, *tris*(4-formylphenyl)amine loaded MCM-41and **MCM-TFM** are given in Fig. 2. The corresponding results for the samples derived from SBA-15 along with **SBA-TFM** are shown in supplementary information, Fig. S1. The samples of both the series show hexagonal 2D-ordering of mesopores as indicated by the presence of three distinct diffraction peaks corresponding to 100, 110 and 200 planes with a weak signal for 210^27^. However, with functionalization, the peaks shift slightly to higher 2θ values along with decrease in intensity. This indicates reduction in pore size as well as ordering of the pores to some extent. The diffraction patterns of the probes, **MCM-TFM** and **SBA-TFM**, obtained by grafting 2-aminothiophenol *via* Schiff base condensation to the functionalized silica supports remain almost unchanged compared to the functionalized supports. This indicates that there is no significant change in the mesostructure after incorporation of 2-aminothiophenol.

### Nitrogen adsorption/desorption studies

**Figure 3:**
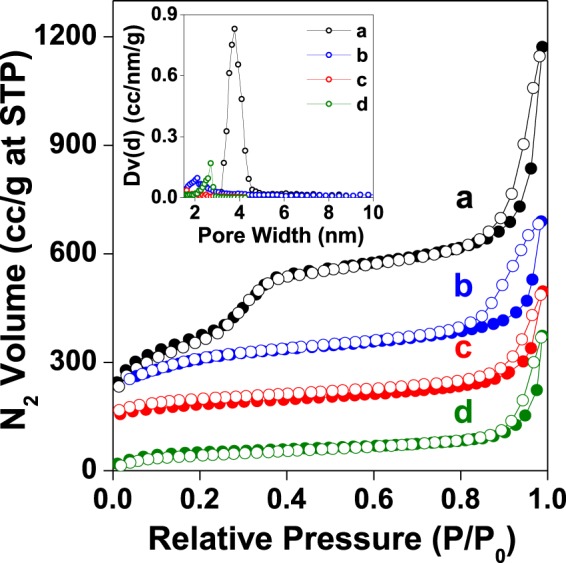
Nitrogen adsorption desorption isotherms of (**a**) calcined MCM-41, (**b**) 3-APTES functionalized MCM-41, (**c**) *tris*(4-formylphenyl)amine loaded functionalized MCM-41 and (**d**) **MCM-TFM**. For clarity, the Y-axis values have been increased by 110 cc/g, 170 cc/g and 120 cc/g for plot a, b and c, respectively. Adsorption data are marked by filled symbols and desorption data by empty symbols. **Inset**: NLDFT Pore size distribution of (**a**) calcined MCM-41, (**b**) 3-APTES functionalized MCM-41, (**c**) *tris*(4-formylphenyl)amine loaded functionalized MCM-41 and (**d**) **MCM-TFM**.

**Figure 4:**
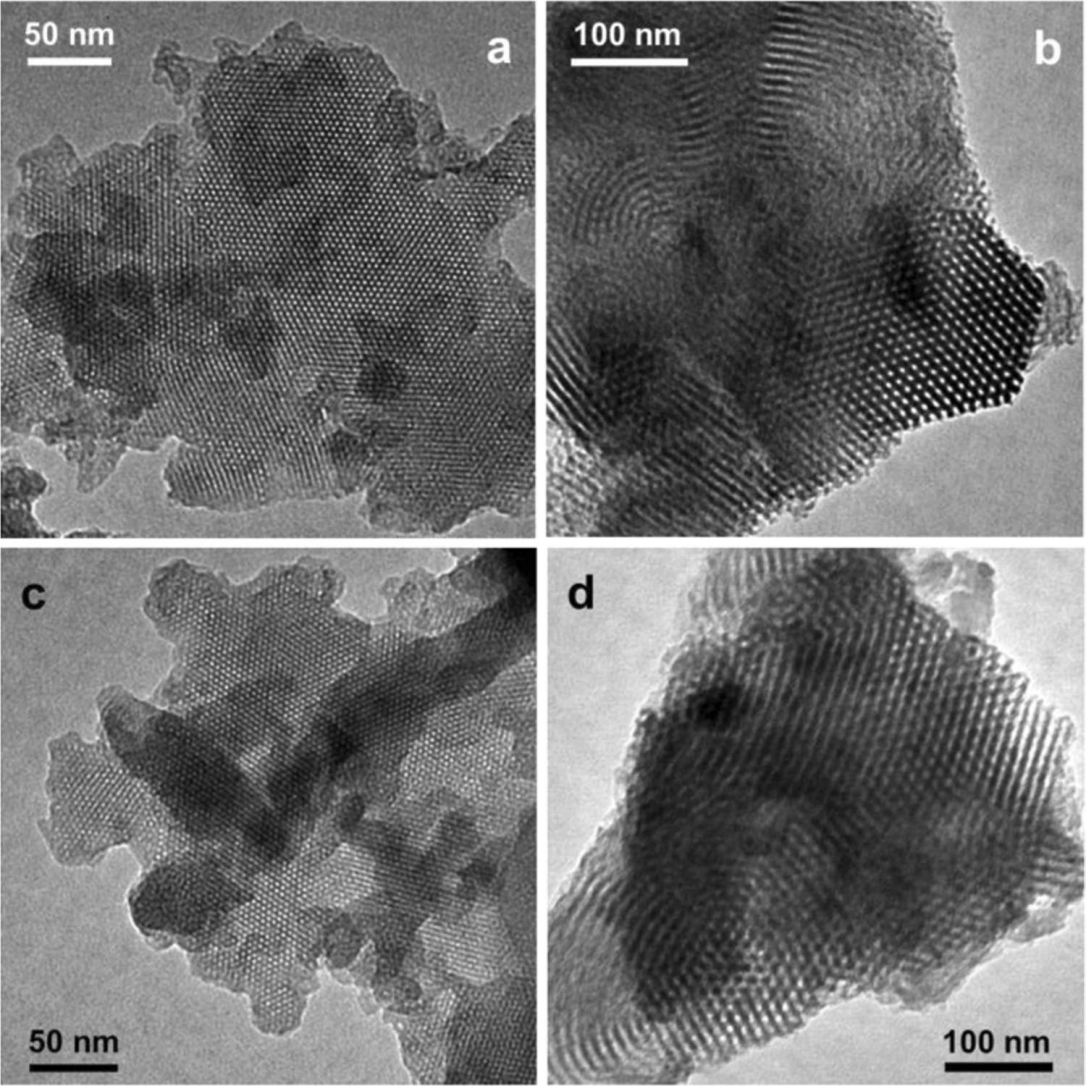
TEM images of (**a**) *tris*(4-formylphenyl)amine loaded MCM-41, (**b**) *tris*(4-formylphenyl)amine loaded SBA-15, (**c**) **MCM-TFM** and (**d**) **SBA-TFM**.

The nitrogen adsorption/desorption studies of all the samples synthesized from two different silica supports, MCM-41 and SBA-15, varying in their pore sizes have been carried out. The isotherms for MCM-41 series of samples including **MCM-TFM** are given in Fig. 3. The corresponding isotherms for SBA-15 series and **SBA-TFM** are shown in Fig. S2. The BET (Brunauer-Emmett-Teller) surface area, pore size and pore volume of the samples are given in Table S1. It is evident that in both the cases there is a gradual decrease in surface area after each step of functionalization. This takes place due to availability of less amount of surface on the functionalized materials for adsorption of nitrogen gas molecules which become occupied due to successive incorporation of various organic moieties. For both series of samples, typical type IV isotherms are obtained with steep rise at higher pressure attributed to capillary condensation and indicating mesoporous nature of the materials^28^. Desorption hysteresis for MCM-41 series of samples arise from intercrystallite adsorption^29^ whereas for SBA-15 series considerably broad H1-type hysteresis is observed, typical for mesoporous materials having 1D cylindrical channels^30^. The average pore diameter of the silica supports, MCM-41 and SBA-15, obtained using NLDFT (non-local density functional theory) model are *ca*. 3.8 and 7.9 nm, respectively (inset of Figs. 3 and S2). As anticipated, the dimension of the pore gradually decreases as gradual functionalization takes place on the pore walls. This pattern is also observed for the pore volume. It is interesting to find that although the pore sizes of samples prepared on SBA-15 are higher compared to the MCM-41 series, the corresponding pore volumes are lower. The basic character of the isotherms remains the same and show type IV pattern for all the materials. The pore sizes show a descending trend *i.e*. with increase in functionalization pore size decreases. The surface area of the final materials, **MCM-TFM** and **SBA-TFM**, are 171 and 170 m^2^g^−1^, respectively, however the pore sizes are *ca*. 2.6 and 5.9 nm, respectively. Incorporation of various functional groups on the silica surface restricts the access of nitrogen gas molecules on the surface and into the pores of the mesoporous samples. However, the hexagonal arrangement of the pores remains intact in both the cases in spite of consecutive processes of functionalization. This can be confirmed from the transmission electron microscopy (TEM) images of the samples given in Fig. 4.

### Transmission electron microscopy studies

The TEM images of **MCM-TFM** and **SBA-TFM**, obtained after functionalization of MCM-41 and SBA-15 successively with 3-APTES, *tris*(4-formylphenyl)amine and 2-aminothiophenol respectively, have been shown in Fig. 4c,d, respectively. In both the samples regular hexagonal arrangement of pores with different contrast from the pore walls can be observed. The TEM images of the intermediate samples obtained after *tris*(4-formylphenyl)amine is grafted on 3-APTES functionalized MCM-41 and SBA-15 has been illustrated in Fig. 4a,b, respectively. It can be observed from the images that the ordering of the pores is affected to some extent with increase in functionalization, which is not beyond expectation. However, the basic ordering of hexagonal pore structure is mostly retained in both the samples. Thus, from powder X-ray diffraction patterns, gas adsorption studies and TEM image analyses the mesoporous structure of the samples could be confirmed.

### FT-IR spectroscopy and thermogravimetric studies

The FT-IR spectra of MCM-41 and SBA-15 series of samples are shown in supplementary information, Figs. S3 and S4. Spectral patterns for both the series of samples are comparable. A band at *ca*. 1060 cm^−1^ appears for the unfunctionalized silica, MCM-41 and SBA-15 (Figs. S3a and S4a) which corresponds to Si-O-Si moieties and can be observed for all the subsequent materials. A broad band at *ca*. 3700–2930 cm^−1^ appears for the 3-APTES functionalized silica which may be attributed to the presence of amine and methylene groups (Figs. S3b and S4b). After incorporation of *tris*(4-formylphenyl)amine into the silica matrices a band emerges at *ca*. 1644 cm^−1^, characteristic of azomethine group. An additional band is observed at *ca*. 1700 cm^−1^ indicating the presence of free aldehyde group (Figs. S3c and S4c). After Schiff-base condensation between free aldehyde group and 2-aminothiophenol the peak for -CHO group disappears indicating formation of C=N (Figs. S3d and S4d). After treatment with Hg^2+^ ion the band at 1644 cm^−1^ shifts to 1594 cm^−1^, which confirms the coordination of the metal center with the imine nitrogen atom (Figs. S3e and S4e). From these studies, it is evident that the structural features of both the starting silica supports as well as different functionalized frameworks derived from them and the metal bound probes are more or less similar.

Thermal analysis is a useful tool to ascertain the amount of organic loading on silica samples. The results of thermogravimetric studies of the samples prepared from MCM-41 and SBA-15 silica are given in supplementary information, Figs. S5 and S6, respectively. The amount of 3-APTES, *tris*(4-formylphenyl)amine and 2-aminothiophenol loaded on the frameworks have been calculated (details in supplementary information) and found to be *ca*. 0.665, 0.198 and 0.480 mmol/g, respectively, for MCM-41 supported samples. Whereas, the corresponding values for SBA-15 supported samples are 0.648, 0.187 and 0.463 mmol/g, respectively.

### Solid state NMR studies

**Figure 5:**
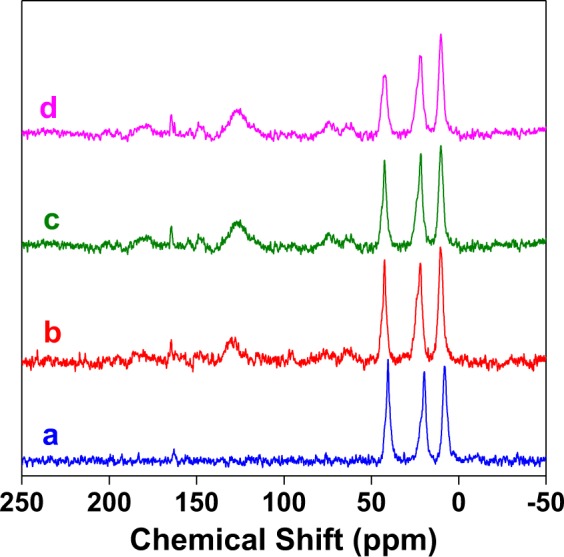
^13^C CP-MAS NMR spectra of (**a**) 3-APTES functionalized MCM-41, (**b**) *tris*(4-formylphenyl)amine loaded MCM-41, (**c**) **MCM-TFM** and (**d**) Hg^2+^-bound **MCM-TFM**.

Solid state MAS NMR studies provide significant information on the structure of the materials, incorporation of organic moieties and metal binding. Spectra of MCM-41 based samples have been shown here which have similar structure as SBA-15 series, as already established from FT-IR studies. While ^29^Si MAS NMR spectra (Fig. S7) clearly shows the change in chemical environment around the silicon atom on organic functionalization, ^13^C CP MAS NMR gives convincing evidence of incorporation of different functional groups (Fig. 5). The ^13^C CP MAS NMR spectrum of 3-APTES linked silica exhibits peaks at 7.9, 19.6 and 40.4 ppm (Fig. 5a) attributed to the aliphatic carbons of aminopropyl units. For *tris*(4-formylphenyl)amine loaded material (Fig. 5b) several new peaks are generated at 10.3, 21.8, 42.3, 61.4, 73.7, 95.2, 96.4, 121.8, 125.9, 127.5, 130.8, 164.8 and 180.5 ppm indicating the presence of aliphatic and aromatic carbons and confirming Schiff-base formation between –NH_2_ of aminopropyl moiety and -CHO of *tris*(4-formylphenyl)amine. The spectra of **MCM-TFM** and Hg-bound **MCM-TFM** are shown in Fig. 5c,d, respectively. These are almost similar to the previous spectrum with identical number of peaks for carbon atoms but with small chemical shifts, indicating retention of structure of the heterogeneous support after condensation and complex formation.

The ^29^Si MAS NMR of calcined MCM-41 shows peaks at *ca*. ‒111.9 and ‒103.1 ppm (Fig. S7a) ascribed to the Q^4^ and Q^3^ silica centers of the Si(OSi)_n_(OH)_4‒n_ moiety. For the 3-APTES functionalized silica (Fig. S7b), in addition to the Q^4^ and Q^3^ silica species additional peaks appear at ‒69.0 and ‒58.3 ppm indicating incorporation of aminopropyl units. Similar kind of hybrid mesoporous silica has been reported in the literature which show chemical shifts at *ca*. 67 and 58 ppm. These peaks may be attributed to the T^3^ ((SiO)_3_Si-R-Si(OSi)_3_) and T^2^ ((HO)_2_(OSi)Si-R-Si(OSi)_2_(OH)) species^31,32^, respectively. The spectra for other samples, including **MCM-TFM** and Hg-bound **MCM-TFM** (Fig. S7c–e), are more or less similar to the 3-APTES grafted sample, indicating the formation of imine bond and its retention after metal binding.

### Sensing and separation of Hg^2+^

**Figure 6:**
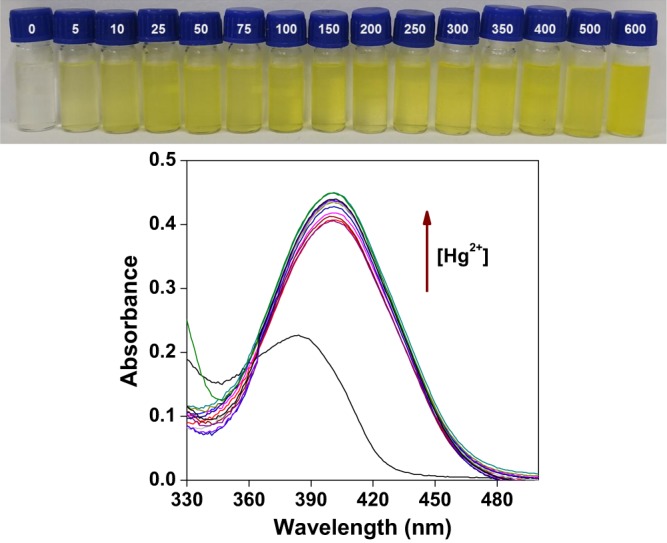
Absorption spectra of **MCM-TFM** (in 14:1 water/THF) in the presence of different concentrations of Hg^+2^ ions (0, 5, 10, 25, 50, 75, 100, 150, 200, 250, 300, 350, 400, 500, 600 μmol/L) at room temperature (Bottom). Visual colorimetric change, concentration in μmol/L (Top).

**Figure 7:**
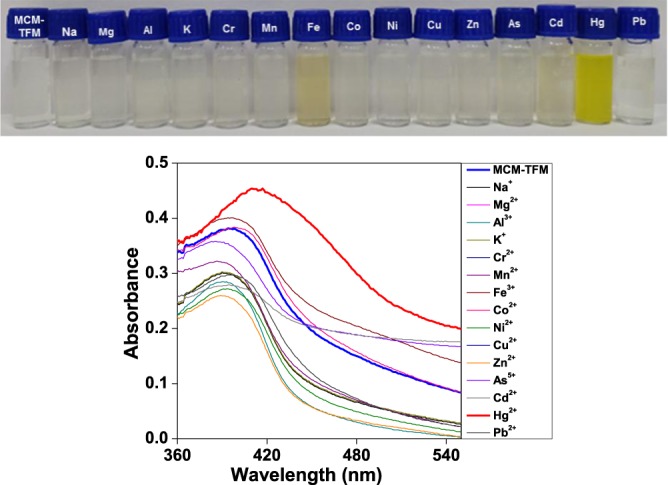
Absorption spectra of **MCM-TFM** (in 14:1 water/THF) in the presence of different metal ions (600 μmol/L) at room temperature (Bottom). Visual colorimetric change (Top).

The mesopore sizes dominant in **MCM-TFM** and **SBA-TFM** and the functional groups incorporated into their frameworks are favourable for adsorption of heavy metal ions. The UV-visible spectra of **MCM-TFM** are recorded in the presence of various concentrations of Hg^2+^ ions in water/THF (14:1) media at room temperature. In absence of Hg^2+^, the probe shows a peak at 384 nm which after addition of Hg^2+^ ions undergoes a red shift to 401 nm. This indicates formation of Hg^2+^-probe complex and the absorption intensity increases with gradual increase in metal ion concentration (Fig. 6). Under identical conditions, other relevant metal ions like Na^+^, Mg^2+^, Al^3+^, K^+^, Ca^2+^, Cr^3+^, Mn^2+^, Fe^3+^, Co^2+^, Ni^2+^, Cu^2+^, Zn^2+^, As^3+^, Cd^2+^ and Pb^2+^ show no shift in peak position (Fig. 7). Thus, the probe, **MCM-TFM** is selective for colorimetric detection of Hg^2+^ only. Similarly, for **SBA-TFM**, Hg^2+^-complex is formed (red shift occurs from 398 to 427 nm) and the absorption intensity increases with increase in metal ion concentration (Fig. S8). However, this material shows somewhat red shift in the spectra with Cd^2+^ and Pb^2+^ solutions as well; though such shifts are less compared to Hg^2+^ (Fig. S9). Also, such shifts do not affect the visual colorimetric change which is prominent only for Hg^2+^ and almost undetectable for the other two metal ions. Nevertheless, it can be said that **MCM-TFM** is a better probe than **SBA-TFM** in terms of selective sensing of Hg^2+^. From UV-visible titration, the saturation concentration and minimum sensing concentration have been found to be 600 μmol/L and 250 ppb, respectively, for both the probes. The colour changes of **MCM-TFM** and **SBA-TFM**, from pale yellow to orange, in the presence of Hg^2+^ can be monitored by naked eyes.

The sensitivity of **MCM-TFM** and **SBA-TFM** for Hg^2+^ has been ascertained by the determination of limit of detection (LOD) values from the absorbance data. LOD has been calculated by 3σ method^33^. Details of LOD calculation are given in supplementary information (Figs. S10–S13). The LOD values of **MCM-TFM** and **SBA-TFM** for Hg^2+^ have been determined to be 6.15 × 10^−5^ and 3.48 × 10^−4^ mol/L, respectively.

To understand the effect of pH on the sensing behaviour of the probes, the absorption spectra of **MCM-TFM** and **SBA-TFM** have been studied under acidic and basic conditions in addition to the experimental pH condition. The experimental solution prepared in double distilled water and containing Hg^2+^ attained a pH of *ca*. 6.2. To study the response of the materials in acidic and basic media the pH of the solutions has been adjusted to approximately 2, 4, 8, 10 and 12 by the addition of acid and base, respectively. The results of the studies have been given in supplementary information, Figs. S14 and S15, for **MCM-TFM** and **SBA-TFM**, respectively. In both the cases, in acidic medium (pH = 2) there is a red shift in the absorption maxima towards higher wavelength with somewhat lowering in intensity. These observations can be attributed to the protonation of = N groups present in the probes to =NH^+^ which results in reduced affinity for and weaker coordination with Hg^2+^ ion^22^. On the other hand, in basic medium (pH = 12) no shifting is observed and the absorption peak position remains unaltered. However, there is a decrease in the intensity of the spectra probably due to prevalence of hydrated species of Hg^2+^, such as Hg(OH)_2_^34^. Thus, under both acidic and basic conditions there is marginal lowering in absorption intensity compared to the experimental condition. Thus, it can be concluded that the probes can be used over a wide range of pH which is an important property for practical applications.

To determine the amount of active sites present in **MCM-TFM** and **SBA-TFM**, macroscopic complexometric analyses have been carried out. For that, known amount of the probes are treated with known concentration of Hg^2+^ solution, and the residual metal concentration has been determined by titration with standard disodium ethylenediamminetetraacetic acid dihydrate (Na_2_EDTA) solution. The amount of Hg^2+^ bound to the samples and hence the active sites present is then calculated and has been found to be 1.15 and 0.92 mmol/g for **MCM-TFM** and **SBA-TFM**, respectively. The stability and effective regeneration of the probes are essential criteria for their practical applications as adsorbents and hence, studies on the reusability of the probes are extremely important. Rapid and efficient metal decomplexation experiments have been carried out using Na_2_EDTA solution for regenerating the adsorbents which are subsequently subjected to next cycle of metal ion adsorption.

The recyclability of **MCM-TFM** and **SBA-TFM** has been investigated by repeating the complexation-decomplexation processes for four times (Table S2). The adsorption capacity hardly changed for the first two cycles. After four successive cycles, the removal efficiencies drop to 76% and 72%, respectively, with respect to the first cycle. The adsorption capacity remains still high. These data clearly demonstrate that the silica supported probes have remarkable regeneration capability even after multiple uses. The surface areas of the samples before and after metal binding in four consecutive cycles are given in Table S3. The results show that after Hg^2+^-adsorption the surface area of the probe decreases and on removal of the metal ion by chelation the surface area is regained. However, this recovery is not 100% and the net surface area of the adsorbents decrease in every cycle. Further, time dependent adsorption studies show that adsorption capacity of **MCM-TFM** reaches 74 and 92% within 15 and 30 minutes of equilibration whereas the corresponding values for **SBA-TFM** are 65 and 80%, respectively (Table S4). The saturation mercury uptake capacity is found to be *ca*. 270 and 231 mg/g, respectively, after 2 h.

To study Hg^2+^ removal efficiency of the samples, hydride generation atomic absorption spectroscopic studies have been carried out. Different known concentration of the metal ion solution have been taken and treated with known amount of the probes and the remaining concentrations of Hg^2+^ have been measured. From these data the amount adsorbed and hence the removal efficiency could be calculated (Table S5). It has been found that for solutions ranging from concentration between 50 ppb to 10 ppm, the removal efficacies for **MCM-TFM** are >95%. Comparable results are obtained for **SBA-TFM**. Such high adsorption capacities can be attributed to the affinity of soft sulphur centres for bond formation with soft acid centres, Hg^2+^ ^8^. At a concentration of 100 ppm, both the probes reach their saturation adsorption limit. From these results, the amounts of active sites in **MCM-TFM** and **SBA-TFM** have been calculated (details in supplementary information) to be 0.98 mmol/g and 0.81 mmol/g, respectively, which are in good agreement with the results obtained from complexometric titrimetry. For columns prepared with 0.2 g of **MCM-TFM**, 95.47 and 99.65% removal efficiencies can be reached with 100 ppb and 100 ppm solutions, respectively. However, for **SBA-TFM**, the separation through column is cumbersome due to light weight of the sample that tends to float on the eluting solvent as well as slow mass transfer rate.

### Comparative discussion with some recent works

A comparative study on recent silica based materials^20–23,35–40^ for sensing and/or separation of mercury has been carried out. A summary of some of the important aspects of the materials has been given in supplementary information, Table S6. Most of the reported works involve either separation or sensing. Only a few (entries 1 and 7) including **MCM-TFM** and **SBA-TFM** (entries 11 and 12) show both sensing as well as separation properties. Most of the materials (entries 1, 2, 4–7, 9 and 10) including the probes reported in this work can be recycled, which is the advantage of using mesoporous silica based materials. Colorimetric sensing and hence visual monitoring of Hg^2+^ could be accomplished only with the materials synthesized in this work which is advantageous as such detection does not need any instrument. The sensitivity of detection has not been reported in some of the works. For our system the sensitivity achieved is somewhat less compared to other reported materials (entries 1, 3 and 8), though none of those are spectrophotometric sensing. The uptake capacity of Hg^2+^ is moderate for **MCM-TFM** and **SBA-TFM**, however the values for the present systems are higher in comparison to materials which have been used both for sensing and separation. Thus, colorimetric detection and reasonably high mercury uptake capacity are the uniqueness of the nanomaterials reported in this work.

## Conclusions

In summary, **MCM-TFM** and **SBA-TFM** have been synthesized by post-synthetic functionalization of MCM-41 and SBA-15 silica matrices as solid supports. The materials have S-donor sites anchored on their surface which can selectively detect and sense Hg^2+^ among soft centres like As^3+^, Cd^2+^ and Pb^2+^ and other relevant metal ions. The metal binding can be monitored spectrophotometrically as well as visually through naked eye and the colour change is distinct from yellow to orange. The probes can be used for separation of Hg^2+^ ion in aqueous media and the efficiency is of the order of ppb. The metal ion can be stripped out easily from the adsorbent by treatment with Na_2_EDTA solution. This property has been used to recycle both the adsorbents through complexation-decomplexation equilibria for four cycles. The efficiency of **MCM-TFM** is found to be somewhat better than **SBA-TFM**, both in terms of sensing and separation, and particularly in column separation the former shows very good permeability. This can be a new perspective for removal of Hg^2+^ and other heavy metal ions from polluted water for environmental remediation.

## Methods

### Synthesis of MCM-41 and SBA-15 and their functionalization with (3-aminopropyl)triethoxysilane (3-APTES)

All the chemicals used in the study have been obtained commercially. The company name and purification percentage of the chemicals and solvents have been given in supporting information, Table S7.

MCM-41 was synthesized as follows^41^. 0.78 g of tartaric acid was dissolved in 60 mL of water and 2.96 g of cetyltrimethylammonium bromide (CTAB) (8.14 × 10^−3^ mol) and 1.5 g of Brij-35 (C_12_H_25_ (OC_2_H_4_)_23_OH, a polyether and aliphatic hydrocarbon chain surfactant) were added to the acidic aqueous solution under stirring at room temperature for 0.5 h. To it, 3.5 g of tetraethylorthosilane (TEOS; 16.8 × 10^−3^ mol) was added drop wise and the mixture was stirred for 2 h. Then 1 (M) aqueous NaOH solution was added to the mixture until the pH reached *ca*. 11. The resulting white mixture was kept under stirring for 12 h at room temperature and then treated hydrothermally at 353 K for 72 h under static condition. The product was separated by filtration, washed thoroughly with water and dried under vacuum. The resulting white powder was subsequently calcined at 723 K for 8 h to remove the organic surfactants.

For the synthesis of SBA-15^42^, 1.7 g of Pluronic P123 (poly(ethylene glycol)-blockpoly(propylene glycol)-block-poly(ethylene glycol)) was dissolved in 62 ml of water under stirring. Then 5.2 mL of 35% HCl was added to it followed by drop wise addition of 3.5 g of TEOS. The mixture was stirred at 313 K for 20 h and the resulting white gel was kept at 373 K for 20 h without stirring. Then the mixture was filtered and the white solid was washed repeatedly with water and ethanol and dried at 373 K. The mesoporous silica was then obtained as a white powder after calcination at 773 K for 10 hours in a flow of air.

MCM-41 and SBA-15 were functionalized with 3-APTES by stirring 1.0 g of each of the material with 1.5 g of 3-APTES for 12 h in chloroform medium at room temperature under nitrogen atmosphere. The white products were obtained by filtration followed by washing repeatedly with chloroform and dichloromethane and finally drying in air.

### Synthesis of *tris*(4-formylphenyl)amine and its grafting on 3-APTES functionalized MCM-41 and SBA-15

The trialdehyde, *tris*(4-formylphenyl)amine was synthesized through Vilsmeier-Haack formylation reaction of triphenylamine (Fig. S16)^43^. In a typical synthesis, 9.5 mL of phosphorus oxychloride was cautiously added to 7.26 mL of dimethyl formamide taken in a round bottom flask, maintaining the temperature at 273 K. The resulting dark yellow viscous liquid (iminium salt) was stirred for 1 h and 1.0 g of triphenylamine was added to the mixture under stirring for 4 h at 368 K. The resulting brown mixture was cooled to room temperature, poured into 200 mL of ice cold water and made alkaline by adding 1(M) NaOH solution. The organic layer was then separated by washing repeatedly with dichloromethane (DCM) and water. The collected organic part was filtered over a short pad of silica gel and dried with Na_2_SO_4_. The solvent was removed by rotary evaporation and to the residue ice cold mixture of 5.78 mL dimethyl formamide and 7.26 mL phosphorus oxychloride was added. The solution was stirred for 1.5 hours at 368 K, cooled to room temperature, poured into ice cold water and made alkaline with 1(M) NaOH solution. The organic layer was isolated by washing with DCM, water and brine solution and then dried using Na_2_SO_4_. After evaporation of the solvent the crude product was purified by column chromatography (using DCM as solvent). The formation of the desired product was confirmed from NMR data (Fig. S17). Yield = 46%, M.P. = 506–508 K.

To graft the trialdehyde on the silica supports, 1.0 g of 3-APTES functionalized MCM-41 or SBA-15 were refluxed with 0.59 g (1.8 milimole) of *tris*(4-formylphenyl)amine dissolved in methanol for 4 h (Fig. S18). The mole ratio of amine to trialdehyde was maintained at 1:1 so that only one formyl group undergoes Schiff base condensation with the amine group of 3-APTES and the other two formyl groups in each molecule remains unreacted. The resulting light yellow solids were isolated by filtration, washed repeatedly with methanol until the filtrate became colorless and dried in a vacuum desiccator.

### Synthesis of **MCM-TFM** and **SBA-TFM**

The *tris*(4-formylphenyl)amine grafted MCM-41 or SBA-15 obtained from the previous step were treated with 0.45 g (3.6 milimole) of 2-aminothiophenol which undergo Schiff base condensation with the two residual –CHO groups of the trialdehyde (Fig. 1). 2-aminothiophenol was taken two times with respect to *tris*(4-formylphenyl)amine and the suspensions of silica supports were stirred for 10 h at room temperature in methanol medium. The yellow solids, **MCM-TFM** and **SBA-TFM**, were isolated by filtration, washed with methanol and dried under vacuum^32^.

### Spectrophotometric studies

For sample preparation, 20 mg of the probe, **MCM-TFM** or **SBA-TFM** have been dispersed in 20 ml of tetrahydrofuran (THF) by sonication for 15 minutes. Then 0.2 ml of this solution has been mixed with different volumes of aqueous Hg^2+^ stock solution (3.0 mmol/L). The total volumes of all the solutions are made up to 3 ml and the final concentrations of the solutions have been varied from 0 to 600 μmol/L. The ratio of water/THF (v/v) in the final dispersion is 14:1. Absorption spectra of **MCM-TFM** and **SBA-TFM** in the presence of different concentrations of Hg^+2^ ions at room temperature are collected with these solutions.

### Complexometric titrimetry to study adsorption capacity of Hg^2+^ and recyclability of the probes

The amount of Hg^2+^ captured by **MCM-TFM** and **SBA-TFM** are estimated by titrimetric method using disodium ethylenediamminetetraacetic acid dihydrate (Na_2_EDTA) and Eriochrome black T (EBT) indicator. The recyclability and reversibility has also been studied by this technique. First, 0.01 M Na_2_EDTA solution has been prepared and its strength is determined against primary standard solution, 0.01 M zinc acetate. The standardized Na_2_EDTA solution is then used to find the exact strength of a 0.01 M HgCl_2_ solution which has been used for all further studies as the source of Hg^2+^. To study the adsorption capacity of the probes, 25 ml of the Hg^2+^ stock solution is allowed to equilibrate with 0.05 g of **MCM-TFM** or **SBA-TFM** for 30 min. The mixture is filtered and 50 ml of 0.01 M Na_2_EDTA solution is added to the filtrate. A portion of the solution forms stable complex with Hg^2+^ present in solution and the excess Na_2_EDTA is estimated by back titration using standard zinc acetate solution. The amount of Hg^2+^ consumed by the probes and hence the amount of active sites present in per gram of the probes is calculated from the results. To check the reversibility of the probes, the solid residues containing adsorbed Hg^2+^ are stirred with 25 ml standardized Na_2_EDTA solution, filtered and the filtrates are back titrated using standard zinc acetate solution. The amount of Hg^2+^ recovered from the probes is calculated. The residues remaining after stripping off the metal ions is washed, dried and used for the next cycle in a similar way. Likewise, the probes have been used up to four consecutive cycles to check their recyclability. Along with the complexation/decomplexation capacities, the surface areas of the probes before and after metal complexation in each cycle have been measured. To study the time-dependent adsorption capacity the probes are allowed to equilibrate with Hg^2+^ stock solution for 15 min, 30 min, 1 h and 2 h, respectively, and analyzed in a similar way.

### Atomic Absorption Spectroscopic (AAS) measurement to study Hg^2+^ removal efficiency

Hydride generation atomic absorption spectroscopic studies have been used to study Hg^2+^ removal efficiency. For all atomic absorption spectroscopic measurements, 0.005 g of the probes are treated with 15 ml of the corresponding standard solutions as mentioned in Table S5. AAS data shows that both **MCM-TFM** and **SBA-TFM** reach their saturation adsorption value with 100 ppm of Hg^2+^ solution when treated with 0.005 g of the respective probes. From this the active binding sites present in the materials can be calculated.

### Instrumental

Powder X-ray diffraction patterns have been collected on a Bruker D-8 Advance instrument, Germany, using Ni-filtered Cu-Kα radiation (λ = 1.5406 Å) working at 40 kV and 40 mA^18,44^. Nitrogen sorption isotherms are measured at 77 K using a NOVA 2200e surface area and pore size analyzer, Quantachrome Instruments, USA. Prior to the measurements, the samples are degassed for 8–12 h at 323, 393 or 423 K depending on the nature of the framework. The specific surface areas are obtained from the isotherms using Brunauer-Emmett-Teller (BET) method and the pore size distribution profiles from non-local density functional theory (NLDFT) model. Transmission electron microscopy (TEM) studies have been performed in a JEOL JEM-1400 transmission electron microscope, Japan. Sample grids have been prepared by putting one drop of the samples dispersed in ethanol on a thin layer of amorphous carbon coated 400 mesh copper grid. FT-IR spectra have been recorded on a PerkinElmer spectrometer (Spectrum Two, USA) using attenuated total reflectance (ATR) technique. Solid state ^13^C CP and ^29^Si MAS NMR spectra are recorded in a CHEMAGNETICS 300 MHz CMX 300 spectrometer, USA. Thermogravimetric studies have been carried out under nitrogen atmosphere (flow rate: 50 cc/min) from room temperature to 1073 K (heating rate of 2 K/min) in a PerkinElmer STA -6000 thermal analyzer, USA. UV-visible spectral studies have been carried out using a Shimadzu UV-2450 spectrophotometer, Japan. For atomic absorption spectroscopy studies using hydride generation technique PerkinElmer PinAAcle 900F, USA, has been used. ^1^H NMR spectrum is recorded in CDCl_3_ solvent using a Bruker 400 MHz spectrometer, Germany, with tetramethylsilane (δ = 0) as the internal standard. The chemical shifts are expressed in parts per million (δ).

## Supplementary information

Supplementary information

## Data Availability

The authors confirm that data supporting the findings of this study is available to Editorial Board Members and referees for the purpose of evaluating the manuscript.
