# Supplementary material for: Mesoporous silica based recyclable probe for colorimetric detection and separation of ppb level Hg2+ from aqueous medium
Source: Sci Rep. 2019 Dec 18;9:19378. doi: 10.1038/s41598-019-55910-4 (PMC6920407; doi:10.1038/s41598-019-55910-4)
Supplement: Supplementary file 1 — Supplementary information [file 41598_2019_55910_MOESM1_ESM.pdf]

Supplementary Information

Mesoporous silica based recyclable probe for colorimetric detection and separation of ppb level Hg^{2+} from aqueous medium

Trisha Das, Debdas Singha, Ananya Pal and Mahasweta Nandi*

Integrated Science Education and Research Centre, Siksha Bhavana, Visva-Bharati, Santiniketan 731235, India

E-mail: mahasweta.nandi@visva-bharati.ac.in

ORCID iD: 0000-0002-3114-4774

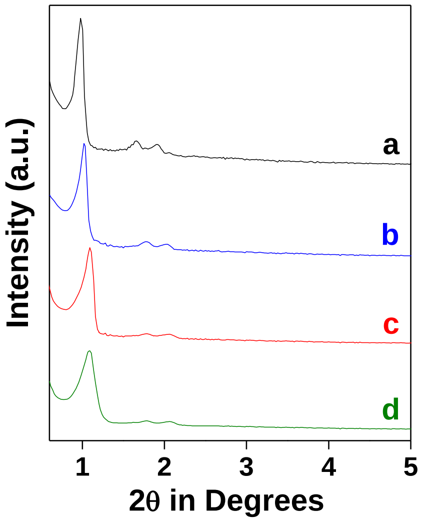

Figure S1. Powder X-ray diffraction patterns of (a) calcined SBA-15, (b) 3-APTES functionalized SBA-15, (c) *tris*(4-formylphenyl)amine loaded SBA-15 and (d) **SBA-TFM**

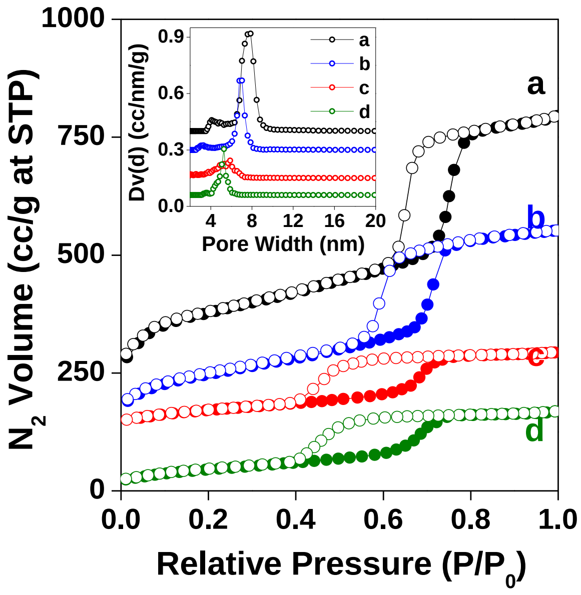

Figure. S2. Nitrogen adsorption desorption isotherms of (a) calcined SBA-15, (b) 3-APTES functionalized SBA-15, (c) *tris*(4-formylphenyl)amine loaded functionalized SBA-15 and (d) **SBA-TFM**. For clarity, the Y-axis values have been increased by 170 cc/g, 130 cc/g and 125 cc/g for plot a, b and c, respectively. Adsorption data are marked by filled symbols and desorption data by empty symbols

Inset: NLDFT Pore size distribution of (a) calcined SBA-15, (b) 3-APTES functionalized SBA-15, (c) *tris*(4-formylphenyl)amine loaded functionalized SBA-15 and (d) **SBA-TFM**

Table S1: Surface area, pore volume and pore sizes of the samples

No.	Sample	BET surface area (m^2g^{-1})	Pore volume (cc/g)	Pore size (nm)
(a)	MCM-41	974	1.63	3.77
(b)	3-APTES functionalized MCM-41	511	0.80	2.70
(c)	*tris*(4-formylphenyl)amine grafted on 3-APTES MCM-41	249	0.59	2.12
(d)	**MCM-TFM**	171	0.57	2.12
(e)	SBA-15	709	0.965	7.81
(f)	3-APTES functionalized SBA-15	425	0.652	6.92
(g)	*tris*(4-formylphenyl)amine grafted on 3-APTES SBA-15	239	0.25	5.880
(h)	**SBA-TFM**	170	0.26	5.26

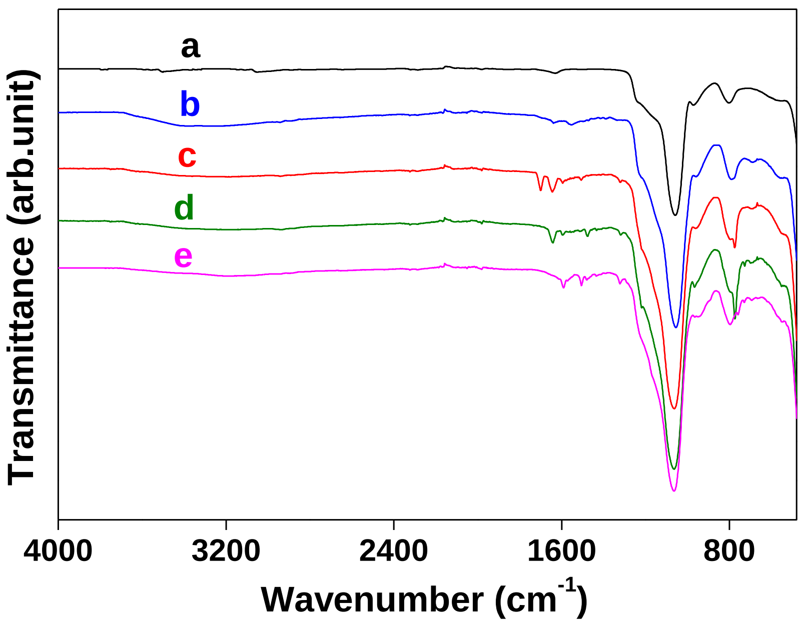

Figure S3. FT-IR spectra of (a) calcined MCM-41, (b) 3-APTES grafted MCM-41, (c) *tris*(4-formylphenyl)amine functionalized MCM-41, (d) **MCM-TFM** and (e) Hg-bound **MCM-TFM**

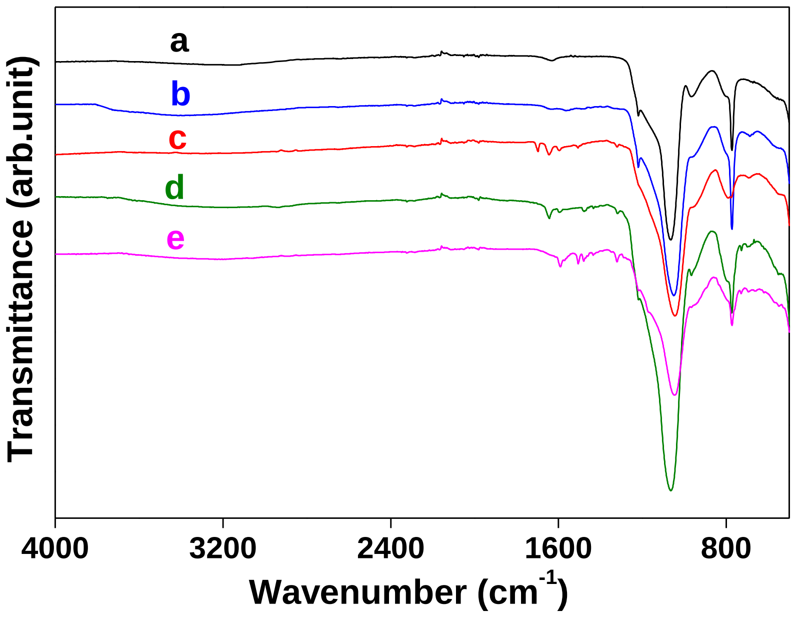

Figure S4. FT-IR spectra of (a) calcined SBA-15, (b) 3-APTES grafted SBA-15, (c) *tris*(4-formylphenyl)amine functionalized SBA-15, (d) **SBA-TFM** and (e) Hg-bound **SBA-TFM**

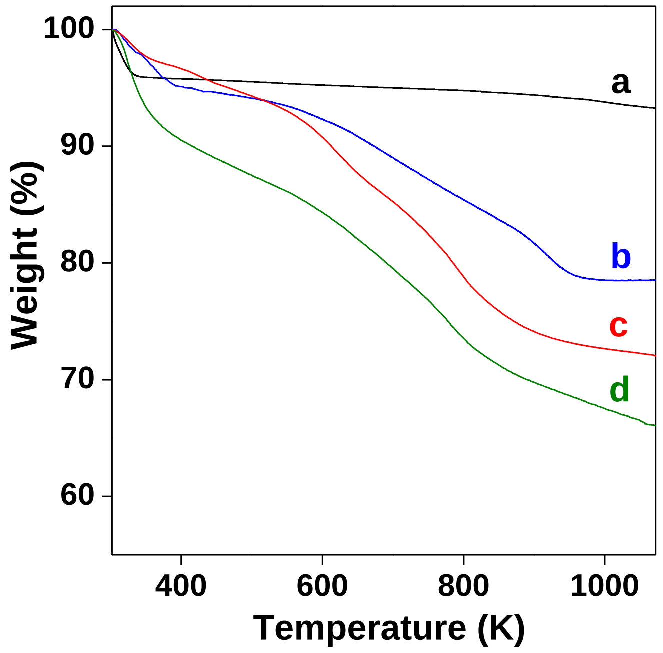

Figure S5. Thermogravimetric analyses of (a) calcined MCM-41, (b) 3-APTES grafted MCM-41, (c) *tris*(4-formylphenyl)amine functionalized MCM-41 and (d) **MCM-TFM**

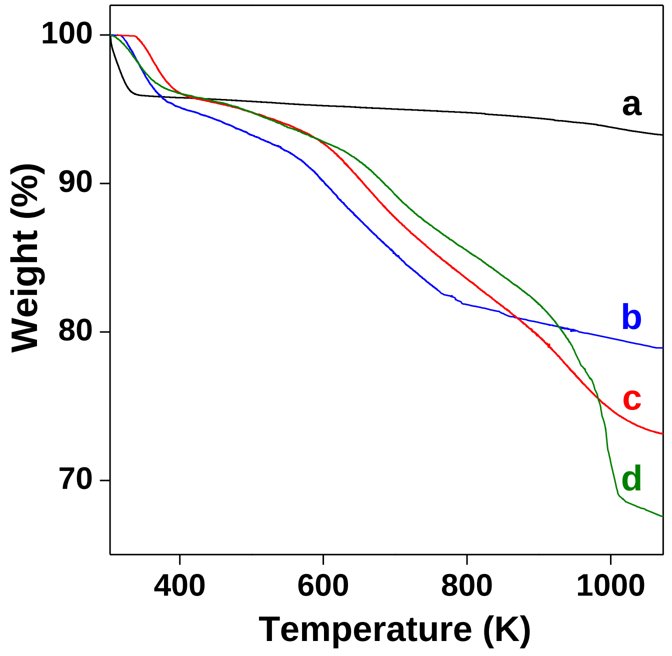

Figure S6. Thermogravimetric analyses of (a) calcined SBA-15, (b) 3-APTES grafted SBA-15, (c) *tris*(4-formylphenyl)amine functionalized SBA-15 and (d) **SBA-TFM**

Calculation of the amount of organic functionalization from thermogravimetric analyses

The amounts of functionalization at various steps have been calculated in the following way.

1. Loading of 3-APTES

5.305 mg of 3-APTES functionalized MCM-41 has been taken for the analysis.

From the difference in weight obtained from the plot in Fig. S5a and b, we find (93.26 – 78.56) = 14.7 mg weight loss has taken place for 100 mg sample due to 3-APTES grafting.

So, for 100 mg weight loss is 14.7 mg.

Hence, for 5.305 mg weight loss is 14.7×5.305/100 mg = 0.779 mg

This is equivalent to 0.779/221.37 = 0.0035 mmol of 3-APTES [Mol. Wt. of 3-APTES = 221.37 g/mol]

Thus, 5.305 mg sample contains 0.0035 mmol of 3-APTES

i.e., 1 g (1000 mg) of the sample (MCM-41) contains 0.00352×1000/5.305 = 0.66 mmol of 3-APTES

2. Loading of *tris*(4-formylphenyl)amine

8.219 mg of *tris*(4-formylphenyl)amine loaded MCM-41 has been taken for the analysis.

From the difference in weight obtained from the plot in Fig. S5b and c, it can be calculated (78.56 – 72.02) = 6.54 mg weight loss takes place for 100 mg sample due to *tris*(4-formylphenyl)amine loading.

So, for 100 mg weight loss is 6.54 mg.

Hence, for 8.219 mg weight loss is 0.537 mg which is equivalent to 0.537/329 = 0.00163 mmol of *tris*(4-formylphenyl)amine [Mol. Wt. of *tris*(4-formylphenyl)amine = 329 g/mol]

Thus, 8.219 mg of sample contains 0.00163 mmol of *tris*(4-formylphenyl)amine

i.e., 1 g of the sample contains 0.00163×1000/8.219 = 0.198 mmol of *tris*(4-formylphenyl)amine

3. Loading of 2-aminothiophenol

5.228 mg of **MCM-TFM** has been taken for the analysis.

From the difference in weight obtained from the plot in Fig. S5c and d, we find (72.02 – 66.07) = 5.95 mg weight loss takes place for 100 mg sample due to binding with 2-aminothiophenol.

So, for 100 mg weight loss is 5.95 mg.

Hence, for 5.228 mg weight loss is 0.312 mg which is equivalent to 0.312/125.19 = 0.00249 mmol of 2-aminothiophenol [Mol. Wt. of 2-aminothiophenol = 125.19 g/mol]

Hence, 5.228 mg of **MCM-TFM** contains 0.00249 mmol 2-aminothiophenol

i.e., 1 g of **MCM-TFM** contains 0.00249×1000/5.228 = 0.48 mmol 2-aminothiophenol

In similar way, the amounts of functionalization have been calculated for SBA-15 series of samples.

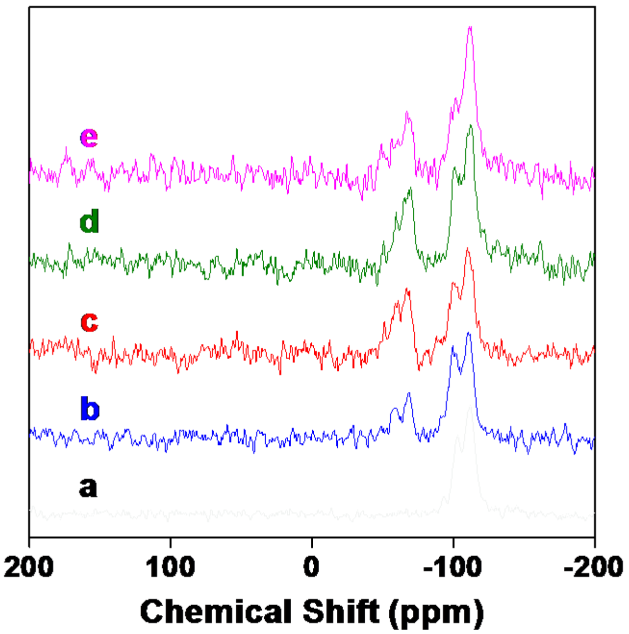

Figure S7. Solid state ^{29}Si MAS NMR spectra of (a) calcined MCM-41, (b) 3-APTES grafted MCM-41, (c) *tris*(4-formylphenyl)amine functionalized MCM-41, (d) **MCM-TFM** and (e) Hg-bound **MCM-TFM**

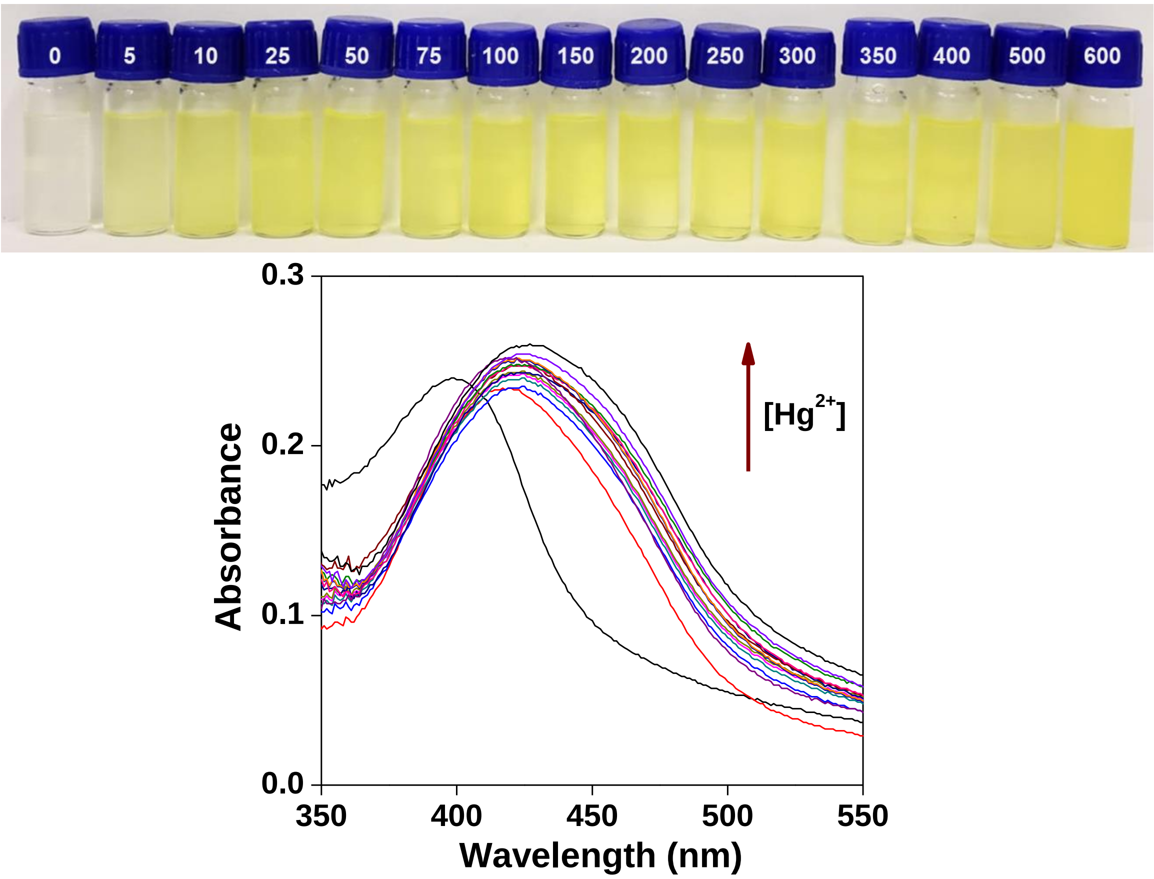

Figure S8. Absorption spectra of **SBA-TFM** (in 14:1 water/THF) in the presence of different concentrations of Hg^{+2} ions (0, 5, 10, 25, 50, 75, 100, 150, 200, 250, 300, 350, 400, 500, 600 μmol/L) at room temperature (Bottom). Visual colorimetric change, concentration in μmol/L (Top)

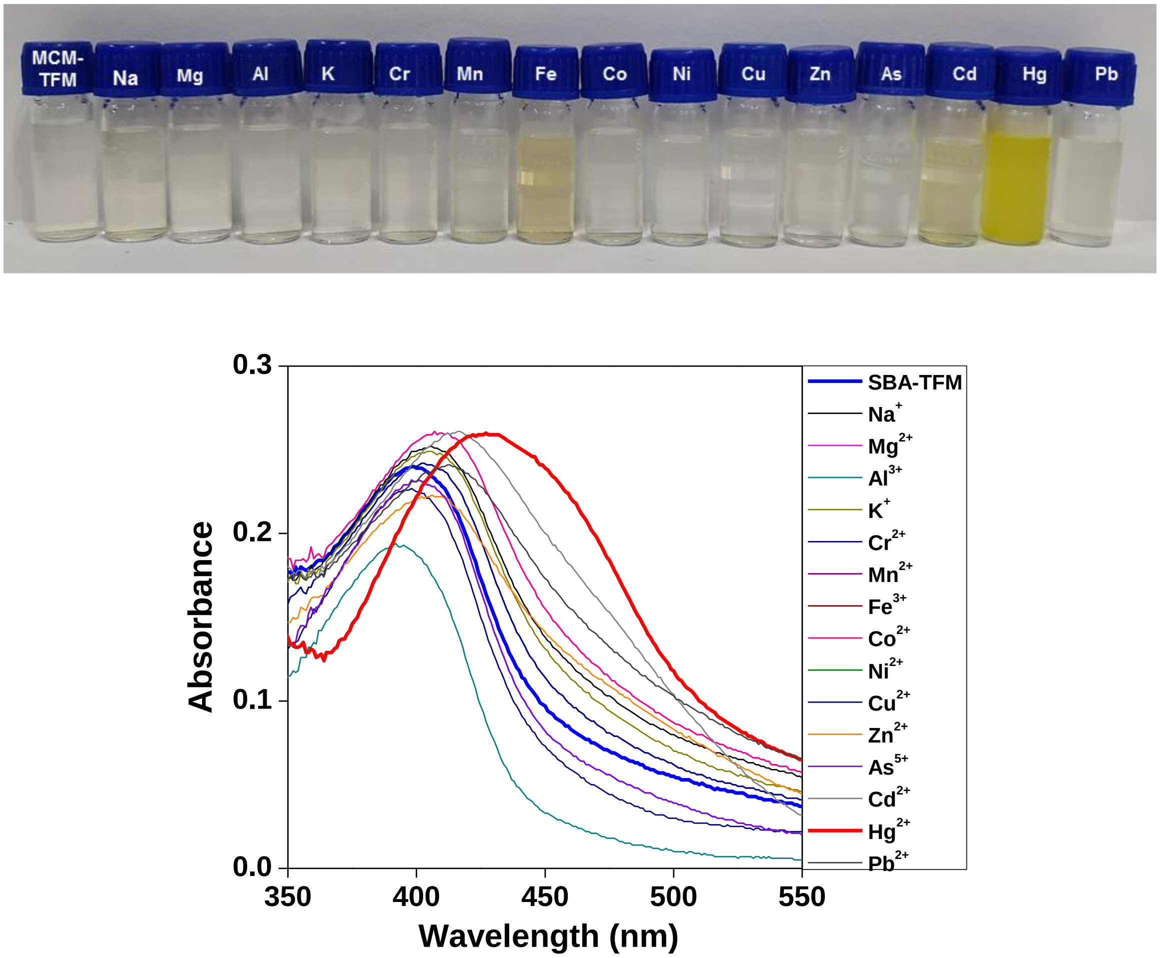

Figure S9. Absorption spectra of **SBA-TFM** (in 14:1 water/THF) in the presence of different metal ions (600 μmol/L) at room temperature (Bottom). Visual colorimetric change (Top)

Limit of detection (LOD) for **MCM-TFM** and **SBA-TFM** has been determined by 3σ method using the equation: DL = K* Sb1/S, where K = 2 or 3 (3 in this case). Sb1 is the standard deviation of the blank solution (Figure S10 and S12) and S is the slope of the calibration curve obtained from linear dynamic plots of absorbance *vs*. [Hg^{2+}] in mg/L (Figure S11 and S13).

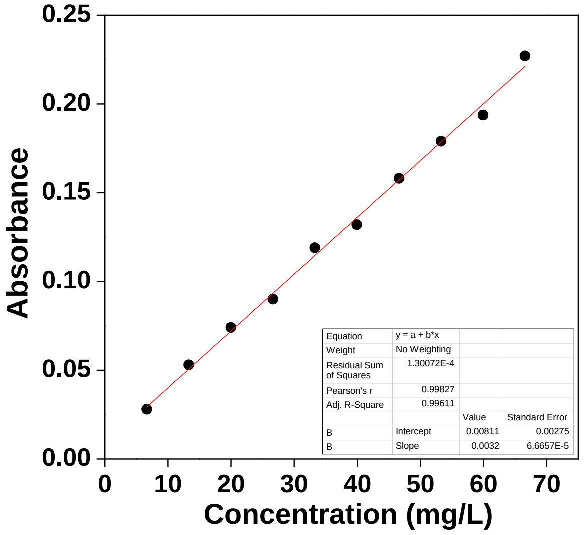

Figure S10. Determination of Sb1 of the blank, **MCM-TFM** in solution

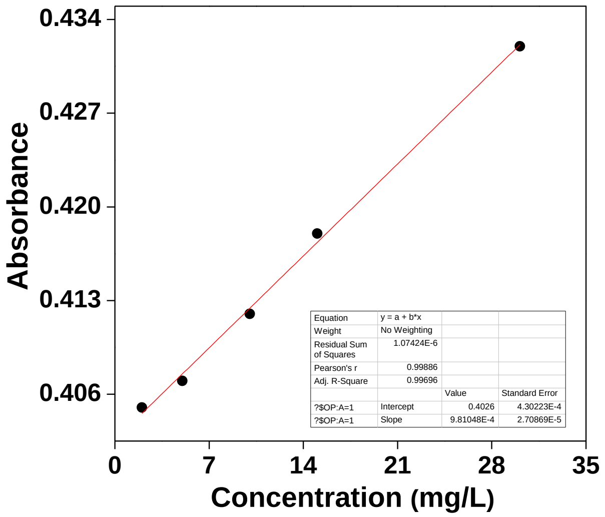

Figure S11. Linear dynamic plot of absorbance *vs*. [Hg^{2+}] for the determination of S (slope) for **MCM-TFM**

Standard deviation (SD) = 0.004032

LOD for **MCM-TFM** = 3 × 0.004032/(9.810 × 10^{-4}) = 12.33 mg/L = 6.15 × 10^{-5} mol/L

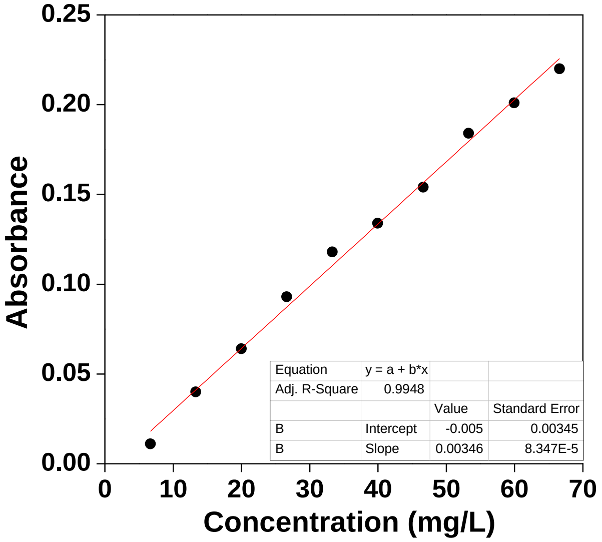

Figure S12. Determination of Sb1 of the blank, **SBA-TFM** in solution

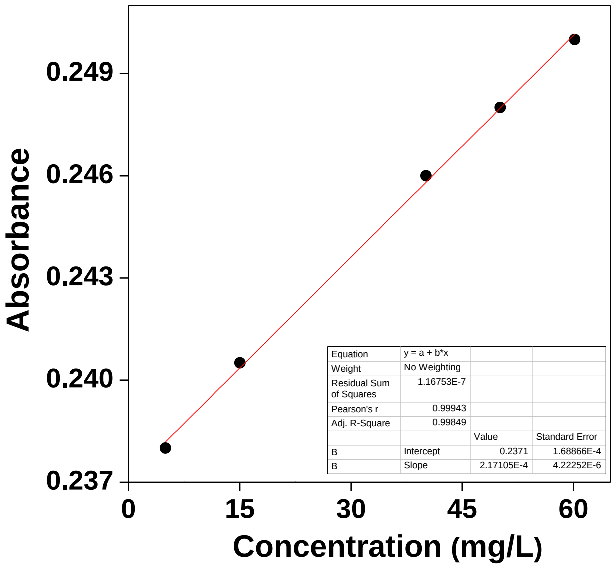

Figure S13. Linear dynamic plot of absorbance *vs.* [Hg^{2+}] for the determination of S (slope) for **SBA-TFM**

Standard deviation (SD) = 0.0050493

LOD for **SBA-TFM** = $3 \times 0.0050493/(2.171 \times 10^{-4}) = 69.77$ mg/L = 3.48×10^{-4} mol/L

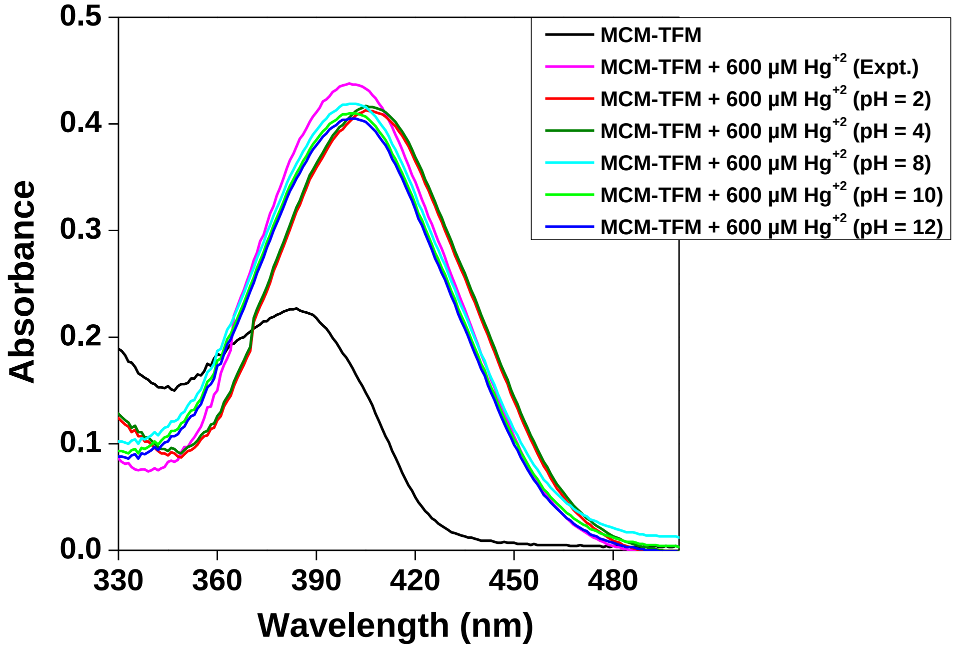

Figure S14. Absorption spectra of **MCM-TFM** (in 14:1 water/THF) at room temperature

- In absence of Hg^{+2} ions
- In presence of 600 μmol/L Hg^{+2} ions at experimental pH
- In presence of 600 μmol/L Hg^{+2} ions at pH = 2
- In presence of 600 μmol/L Hg^{+2} ions at pH = 4
- In presence of 600 μmol/L Hg^{+2} ions at pH = 8
- In presence of 600 μmol/L Hg^{+2} ions at pH = 10
- In presence of 600 μmol/L Hg^{+2} ions at pH = 12

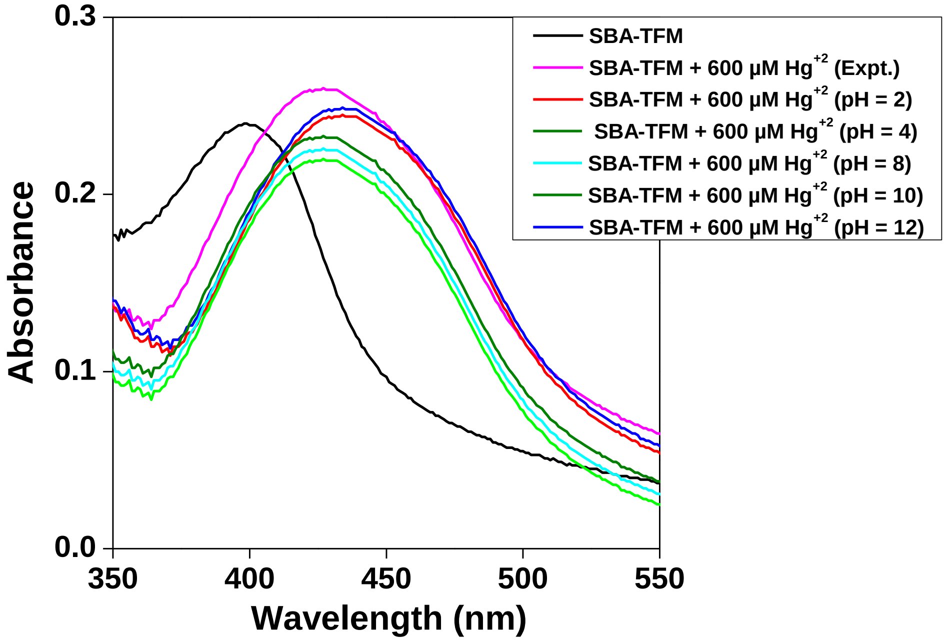

Figure S15. Absorption spectra of **SBA-TFM** (in 14:1 water/THF) at room temperature

- In absence of Hg^{+2} ions
- In presence of 600 μmol/L Hg^{+2} ions at experimental pH
- In presence of 600 μmol/L Hg^{+2} ions at pH = 2
- In presence of 600 μmol/L Hg^{+2} ions at pH = 4
- In presence of 600 μmol/L Hg^{+2} ions at pH = 8
- In presence of 600 μmol/L Hg^{+2} ions at pH = 10
- In presence of 600 μmol/L Hg^{+2} ions at pH = 12

Table S2: Amount of Hg^{2+} adsorbed and recovered (in mg) in each cycle*

Name of Probe	Cycle 1		Cycle 2		Cycle 3		Cycle 4	
	Adsorbed	Recovered	Adsorbed	Recovered	Adsorbed	Recovered	Adsorbed	Recovered
**MCM-TFM**	12.35	12.07	11.73	11.13	10.62	9.95	9.39	8.71
**SBA-TFM**	9.23	8.91	8.69	8.15	7.57	7.06	6.65	6.15

*Equilibration time = 30 min, Amount of probe = 0.05 g

Table S3: Surface area (in m^2g^{-1}) of the probes before and after Hg^{2+} binding

	Cycle 1		Cycle 2		Cycle 3		Cycle 4		Recovered after 4th Cycle
	Before	After	Before	After	Before	After	Before	After	
**MCM-TFM**	171	80	163	77	148	68	133	56	98
**SBA-TFM**	170	75	165	69	154	63	139	53	102

Table S4: Adsorption of Hg^{2+} by the probes, adsorption efficiency and recovery with time

Name of Probe	Time	Hg^{2+}Absorbed (in mg)	Adsorption efficiency (mg/g)	Hg^{2+} Recovered (in mg)
**MCM-TFM**	15 minutes	9.92	198.4	9.42
	30 minutes	12.35	247.0	2.07
	60 minutes	13.104	262.0	12.73
	120 minutes	13.48	269.6	12.95
**SBA-TFM**	15 minutes	7.46	149.2	7.12
	30 minutes	9.23	184.6	8.91
	60 minutes	11.02	220.4	10.68
	120 minutes	11.54	230.8	11.08

Amount of probe = 0.05 g

Table S5: Hg^{2+} removal efficiency of the probes from atomic absorption spectrometric studies

Name of Sample	Weight of probe	Concentration of Hg^{2+} solution		Hg^{2+} removed by the probe	Efficiency (%)
		Before treatment with probe	After treatment with probe		
MCM-TFM	0.005 g	50 ppb	1.966 ppb	48.034 ppb	96.1
		100 ppb	3.146 ppb	96.854 ppb	96.9
		500 ppb	8.602 ppb	491.398 ppb	98.3
		1000 ppb	41.130 ppb	958.870 ppb	95.9
		10 ppm	0.258 ppm	9.742 ppm	97.4
		100 ppm*	34.400 ppm	65.600 ppm	65.6
	0.2 g (column)	100 ppb	4.533 ppb	95.467 ppm	95.5
	0.2 g (column)	100 ppm	0.349 ppm	99.651 ppm	99.7
SBA-TFM	0.005 g	50 ppb	1.848 ppb	48.152 ppb	96.3
		100 ppb	2.811 ppb	97.189 ppb	97.2
		500 ppb	23.120 ppb	476.880 ppb	95.4
		1000 ppb	36.880 ppb	963.120 ppb	96.3
		10 ppm	0.315 ppm	9.685 ppm	96.9
		100 ppm*	45.870 ppm	54.130 ppm	54.1

*Saturation reached

Calculation of amount of Hg^{2+} bound to the samples and amount of active sites

From atomic absorption spectroscopic studies

For all measurements, 0.005 g of the probes is treated with 15 ml of the standard solutions (Table S5).

For **MCM-TFM**, saturation reaches with 100 ppm Hg^{2+} solution.

Thus, Hg^{2+} concentration of the solution before treatment with probe = 100.0 ppm

Hg^{2+} concentration of the solution after treatment with probe (0.005 g) = 34.4 ppm

Therefore, Hg^{2+} adsorbed by 0.005 g of **MCM-TFM** = (100.0 – 34.4) ppm = 65.6 ppm

65.6 ppm implies, 1000 ml solution contains 65.6 mg of Hg^{2+}

Hence, 15 ml solution contains 65.6×15/1000 = 0.984 mg Hg^{2+}

So, 0.005 g **MCM-TFM** can adsorb 0.984 mg Hg^{2+}

i.e., 1.0 g **MCM-TFM** can adsorb 0.984/0.005 mg Hg^{2+} = 196.8 mg Hg^{2+} = 196.8/200.59 = 0.98 mmol of Hg^{2+} [Equivalent weight of mercury = 200.59]

Therefore we can conclude **0.98 mmol** of active sites are present in 1.0 g of **MCM-TFM**.

In similar way, the calculation can be done for **SBA-TFM**.

From EDTA titration

A Zn-acetate primary standard solution [strength = 1.0004 (M/100)] is prepared which is used to standardize a Na_2EDTA solution. The strength of the Na_2EDTA solution is found to be 0.9884 [M/100]. Estimation of Hg^{2+} using Na_2EDTA involves back titration process.

1000 ml of 1 M Na_2EDTA ≡ 1000 ml of 1 M Zn-acetate solution ≡ 1000 ml of 1 M Hg^{2+} solution

Therefore, 1000 ml of 1 M Na_2EDTA ≡ 200.59 g of Hg^{2+} (equivalent weight of mercury)

Amount of Hg^{2+} present in 25 ml ~ 0.01 mol/L Hg^{2+} stock solution

25 ml Hg^{2+} solution is mixed with 50 ml of Na_2EDTA and excess Na_2EDTA is back titrated by standard Zn-acetate solution. The titre value = 26.2 mL [of 1.0004 (M/100) Zn-acetate]

Hence, 50 ml Na_2EDTA ≡ 49.4 mL of 1.0004 (M/100) Zn-acetate

Amount of Hg^{2+} in 25 mL M/100 solution = (49.4 – 26.2) ml of 1.0004 (M/100) Zn-acetate

≡ 23.2 ml of 1.0004 (M/100) Zn-acetate

≡ 0.0020059×23.2×1.0004 g Hg^{2+} = 0.0466 g of Hg^{2+}

Amount of Hg^{2+} adsorbed by MCM-TFM

0.05 g of **MCM-TFM** has been treated with 25 mL of Hg^{+2} solution (containing 0.0466 g of Hg^{2+}) and kept for 30 minutes for attaining equilibrium. After that the solution is filtered and washed with water. To the filtrate 50 mL of standardized Na_2EDTA solution is added. Na_2EDTA forms complex with Hg^{+2} present in solution and the remaining Na_2EDTA is estimated using Zn-acetate solution. The titre value is 31.9 mL [of 1.0004 (M/100) Zn-acetate]

Amount of Hg^{2+} present in the filtrate = (49.4 – 31.9) ×0.0020059×1.0004 g = 0.0351 g

Therefore, Hg^{2+}consumed by **MCM-TFM** = (0.0466 – 0.0351) = 0.0115 g = 0.0115/200.59 = 0.0573 mmol

So, 0.05 g **MCM-TFM** contains 0.0573 mmol of Hg^{2+}

1.0 g **MCM-TFM** contains 1.15 mmol of Hg^{2+}

In similar way, the calculation can be done for **SBA-TFM**.

Table S6: Comparison of some aspects of previously reported related works

Entry	Name	Description	Colorimetric Sensing	Sensing/ Separation	Recyclability	LOD	Hg uptake capacity	Ref.
**1.**	QDs-MMS-Rh6G	CdTe quantum dots/Rh6G doped core-shell magnetic mesoporous silica nanocomposites	No	Both	Yes	2.5×10^{-9} mol L^{-1}	17.7 mg g^{-1}	20
2.	SBA-15-SH	Thiol functionalized SBA-15	No	Separation	Yes	---	195.6 mg g^{-1}	21
3.	$(VBYT)Fe_3O_4@SiO_2$	5-(4-Vinylbenzylidene)amino-1,3,4-thiadiazole-2-thiol-$Fe_3O4@SiO_2$	No	Sensing	No	48nM	---	35
4.	MCM-41-TgA	MCM-41 modified by thioglycolic acid	No	Separation	Yes	---	42.8 mg g^{-1}	36
5.	SH-HMSMCS	Thiol functionalized hollow mesoporous silica spheres with magnetic cores	No	Separation	Yes	---	118.6 mg g^{-1}	22
6.	DT-MCM-41	Magnetic di-thio functionalized mesoporous silica nanoparticles	No	Separation	Yes	---	538.9 mg g^{-1}	23
7.	PAAM-NH_2-MCM-41	Poly-amide derivative grafted to mesoporous silica nanoparticles	No	Both	Yes	---	177 mg g^{-1}	37
8.	Functionalized 2-SBA-15	Calixarene with two dansyl fluorophores grafted on SBA-15	No	Sensing	No	3.3×10^{-7} mol/L	---	38
9.	MCM-41BTU	Benzoylthiourea-modified MCM-41	No	Separation	Yes	---	1000 mg g^{-1}	39
10.	MCM-41ATU	1-Allyl-3-propylthiourea modified MCM-41	No	Separation	Yes	---	300 g mg g^{-1}	40
11.	**MCM-TFM**	MCM-41 functionalized with *tris*(4-formyl phenyl)amine and 2-aminothiophenol	Yes	Both	Yes	6.15×10^{-5} mol/L	196.8 mg g^{-1} (AAS)<br>230.68 mg/g (Na_2EDTA)	*This work*
12.	**SBA-TFM**	SBA-15 functionalized with *tris*(4-formyl phenyl) amine and 2-aminothiophenol	Yes	Both	Yes	3.48×10^{-4} mol/L	162.48 mg g^{-1} (AAS)<br>184.54 mg/g (Na_2EDTA)	*This work*

Table S7: Company name and purification percentage of the chemicals and solvents used

	**Name of Chemical**	**Company name (Purity %)**
1.	Tartaric acid	Merck (≥ 99%)
2.	Cetyltrimethylammonium bromide (CTAB)	Loba Chemie (98%)
3.	Brij-35	Loba Chemie (98%)
4.	Tetraethyl orthosilane	Sigma-Aldrich (98%)
5.	Sodium hydroxide (NaOH)	Merck (≥ 97%)
6.	(3-Aminopropyl)triethoxysilane (3-APTES)	Sigma-Aldrich (≥ 98%)
7.	Pluronic P123	Sigma-Aldrich
8.	Hydrochloric acid (HCl)	Merck (≥ 35%)
9.	Chloroform	Merck (≥ 99%)
10.	Triphenylamine	Sigma-Aldrich (98%)
11.	Dimethyl formamide (DMF)	SRL (99.5 %)
12.	Phosphorus oxychloride ($POCl_3$)	Sigma-Aldrich (99.99%)
13.	2-Aminothiophenol	TCI (> 95%)
14.	Methanol	Research Lab Fine Chem. Industry (99%)
15.	Dichloromethane (DCM)	Merck (≥ 99%)
16.	Mercuric chloride ($HgCl_2$)	Merck (≥ 99%)

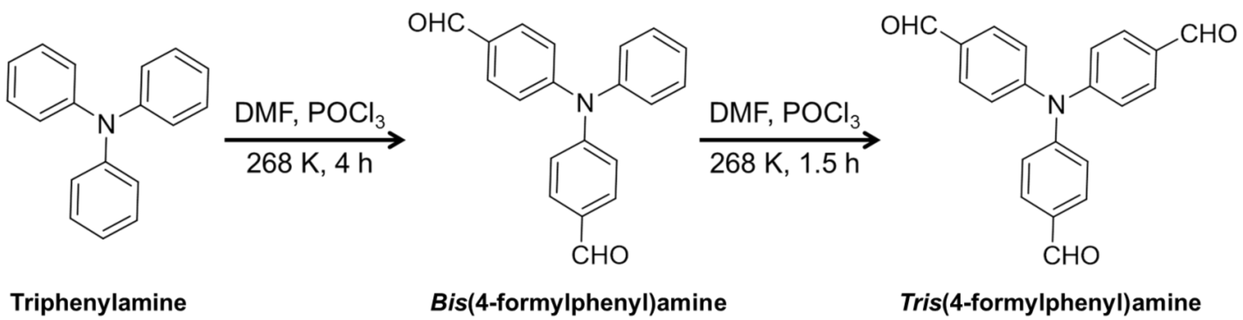

Figure S16. Synthesis of *tris*(4-formylphenyl)amine

***Tris*(4-formylphenyl)amine.** ^{1}H NMR (400 MHz, $CDCl_3$, δ values in ppm): 9.88 (s, 3H), 7.76-7.78 (d, 6H), 7.17–7.19 (d, 6H)

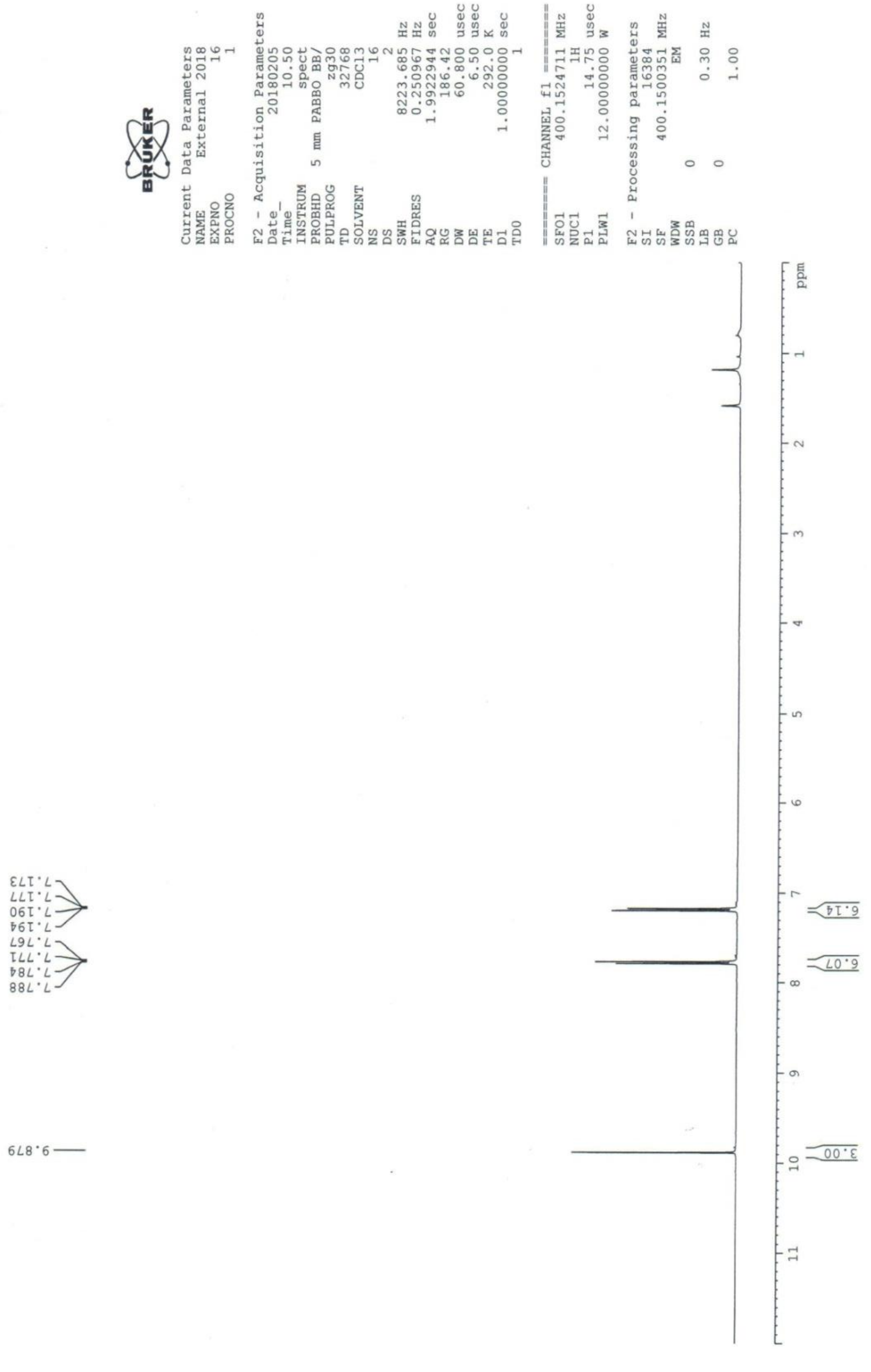

Figure S17. ^{1}H NMR of *tris*(4-formylphenyl)amine

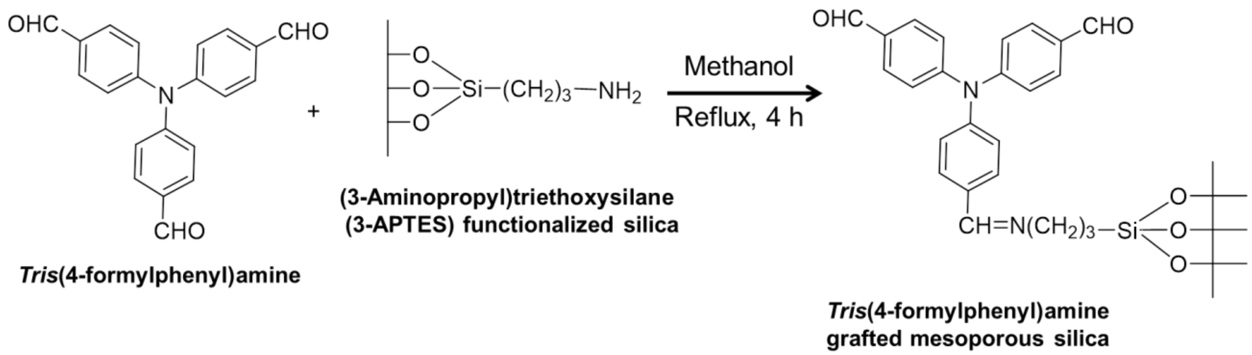

Figure S18. Grafting of *tris*(4-formylphenyl)amine on 3-APTES functionalized MCM-41/SBA-15
